# Assessing and Improving Access to Health and Social Care Services for Children Rendered Vulnerable by Abuse: Protocol for a Cross-Sectoral Longitudinal, Mixed Methods, Multi-Country Study Using Nationwide Data in Europe

**DOI:** 10.23889/ijpds.v11i3.3147

**Published:** 2026-06-11

**Authors:** Catherine Quantin, Donna O’Leary, Yulia Shenderovich, Neha Batura, Marcella Broccia, Jonathan Cottenet, Laura Elizabeth Cowley, Troels Graesholt-Knudsen, Diogo Lamela, Kevin Lalor, Andreas Jud, Leona Hakkaart van Roijen, Peter Fallesen, George Nikolaidis, Athanasios Ntinapogias, Ulugbek Nurmatov, Sinead Brophy, the SERENA Co-Author Group

**Affiliations:** 1 Biostatistics and Bioinformatics (DIM), University Hospital, Bourgogne Franche-Comté University, Dijon, France; 2 INSERM, CIC 1432, Clinical Investigation Centre, Dijon University Hospital, Clinical Epidemiology/Clinical Trials Unit, Dijon, France; 3 Biostatistics, Biomathematics, Pharmacoepidemiology and Infectious Diseases (B2PHI), INSERM, UVSQ, Institut Pasteur, Université Paris-Saclay, Paris, France; 4 Tusla Child and Family Agency, Ireland; 5 School of Applied Social Studies and School of Public Health, University College Cork, Ireland; 6 Wolfson Centre for Young People’s Mental Health, Cardiff University, Cardiff, UK; 7 Centre for Development, Evaluation, Complexity and Implementation in Public Health Improvement (DECIPHer), School of Social Sciences, Cardiff University, Cardiff, UK; 8 Global Business School for Health, University College London, UK; 9 Steno Diabetes Center, Herlev Copenhagen, Denmark; 10 Department of Paediatrics and Adolescent Medicine, Zealand University Hospital, Roskilde, Denmark; 11 Population Data Science, Swansea University Medical School, Swansea University, UK; 12 Department of Forensic Medicine, Aarhus University, Denmark; 13 Lusófona University, Digital Human-Environment Interaction Labs (HEI-Lab), Porto, Portugal; 14 School of Social Sciences, Law and Education, Technological University Dublin, Ireland; 15 School of Social Work, ZHAW Zurich University of Applied Sciences, Zurich, Switzerland; 16 Clinic for Child and Adolescent Psychiatry, Psychosomatics, and Psychotherapy, University of Ulm, Ulm, Germany; 17 Erasmus School of Health Policy & Management, Erasmus Universiteit Rotterdam, Rotterdam, The Netherlands; 18 ROCKWOOL Foundation, Copenhagen, Denmark; Swedish Institute for Social Research, Stockholm University, Stockholm, Sweden; 19 ROCKWOOL Foundation, Copenhagen, Denmark; Swedish Institute for Social Research, Stockholm University, Stockholm, Sweden; 20 Department of Mental Health and Social Welfare, Institute of Child Health, Athens, Greece; 21 Division of Population Medicine, School of Medicine, Cardiff University, Cardiff, UK; 22 National Centre for Population Health and Wellbeing Research, Swansea University, Wales, UK; 23 Health Data Research UK, Wales and Administrative Data Research Wales, Swansea University Medical School, Wales, United Kingdom; † Contributed equally

**Keywords:** Child health, child maltreatment, health and social care, care inequalities, mixed methods, health economics, child protection, child welfare

## Abstract

Child maltreatment (CM) is a widespread and underreported public health concern with long-term health and wellbeing outcomes. In Europe, access to timely, effective support remains limited. Inadequate responses exacerbate long-term outcomes and influence life course trajectories, with substantial societal and economic costs. The EU-funded SERENA project aims to improve access to health and social care (HSC) services for individuals who experience CM throughout Europe by enhancing detection and interventions, limiting consequences, and reducing societal burdens.

SERENA takes an early life course approach, and will examine HSC pathways before and after CM detection, assess related health and wellbeing outcomes, and evaluate the societal costs of CM. Two scoping reviews will examine quantitative and qualitative evidence on barriers and facilitators to access to HSC for children who experience CM, and their service pathways. A mixed methods study will combine quantitative analyses of nationwide longitudinal administrative HSC data from seven countries, supplemented by aggregated child protection data from 26 countries, with qualitative analyses of interviews with adult survivors of CM and HSC professionals in three countries.

Examination of HSC pathways will enable us to identify the settings and stages where interventions can be targeted to improve outcomes for children with CM. We will also examine societal costs by analysing direct medical expenses, educational costs, and productivity losses in four countries. An interdisciplinary, participatory synthesis involving stakeholders and adult survivors of CM will assess services, define priority actions, and inform recommendations.

SERENA, a consortium of 22 partners across Europe, represents the first multi-country, large-scale, cross-sectoral longitudinal initiative to comprehensively examine CM and HSC service use. By addressing critical evidence gaps, SERENA will provide operationally and economically viable recommendations to enhance service access and public health responses in Europe, with findings that are transferable to diverse international contexts.

## Introduction

The World Health Organisation defines child maltreatment (CM) as a major public health concern [1]. Worldwide, an estimated 60% of children less than five years of age experience physical and/or psychological violence by their caregivers, and about 20% of women and 14% of men report childhood sexual abuse [2, 3], with considerable variation between continents and countries [3]. Health and wellbeing depend on the interaction of multiple protective and risk factors, particularly during sensitive and critical periods of development of infants, children, adolescents and youth, and across generations [4]. CM has immediate, cumulative and long-lasting negative effects on development, health and wellbeing [2, 5–7]. These adverse experiences can have social and health consequences that influence life course trajectories, impairing educational achievement and employment, with far-reaching societal and economic impacts [1, 8]. Despite considerable European investments in child protection policies and legal frameworks to safeguard children’s rights [9], CM remains under-identified in health and social care (HSC) systems. For example, among children under five, hospitalisations for physical abuse are estimated at 18/100,000 per year in five European countries [10], although self-report studies estimate the incidence of childhood physical abuse to be much higher [11, 12], with sparse comparative data for other forms of CM. Moreover, social care provision for children who experience CM is poorly understood, with limited insights into how needs evolve beyond childhood, including the provision of adequate support and therapeutic interventions during key life transitions such as entering the educational system, the labour market, and parenthood.

Advancing expertise and driving innovation requires comprehensive data collection and analysis on CM and its associated health, social and economic effects [13, 14]. Multi-country collaboration, with data-sharing and expert networks across multiple sectors and informants - including adult survivors of CM (ASCM) - is essential to address challenges such as the low numbers of identified individuals in administrative registries, inconsistent definitions of CM across sectors and countries, differences in perspectives between professionals and individuals with lived experience, varying data collection practices, limited national guidelines and training, and restricted systematic data linkage [15–18]. Consequently, our understanding of European HSC service provision for CM, including disparities and gaps in access, and long-term effects and service needs, is incomplete, emphasising the need for an expanded surveillance programmes to inform evidence-based policy and practice [3].

Building upon the outcomes and expertise developed through the pan-European initiative Euro-CAN COST Action 19106 (www.euro-can.org)—which laid the groundwork for the SERENA project (Assessing and improving access to health and social care SErvices for children RENdered vulnerable by Abuse)—this project will undertake an early life course approach to identify upstream opportunities and the settings where interventions can be targeted. By doing so, we aim to improve HSC trajectories, support transitions across developmental stages and improve long-term health and wellbeing outcomes for children who experienced CM. SERENA will undertake a critical assessment of current HSC service pathways in multiple European countries. By leveraging in-depth, cross-sectoral data collection and linkage, and incorporating the lived experiences of ASCM, SERENA seeks to identify key structural and procedural barriers to service access, both prior to and following the formal recognition of CM. The overarching aim of SERENA is to enhance access to effective, timely, and contextually appropriate HSC services for individuals impacted by CM in Europe. To this end, the project will generate a set of evidence-based, actionable recommendations spanning childhood and early adulthood life stages at the sectoral, national, and European levels. These recommendations will inform improvements in early detection, the optimisation of care pathways, and ultimately contribute to improved short- and long-term outcomes for affected individuals, based on the rigorous analysis of the consequences of CM during childhood, adolescence and early adulthood, alongside an estimation of its societal and economic burden.

## Methods/Design

### Overall Methodology

**Figure 1:**
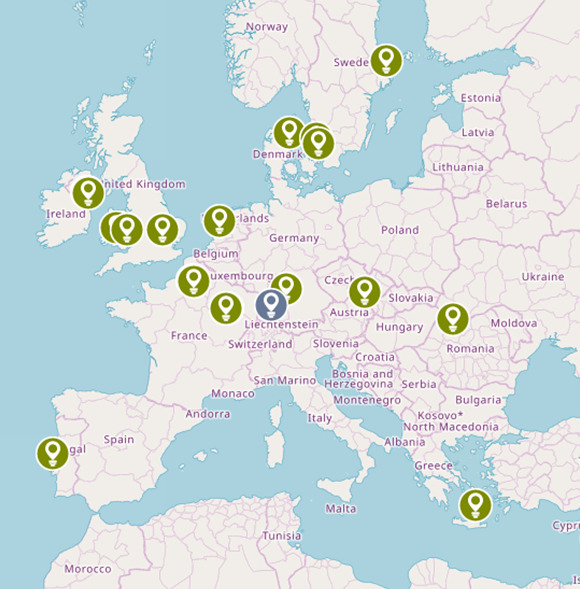
Institutions and Countries Involved in the SERENA Project

**Figure 2:**
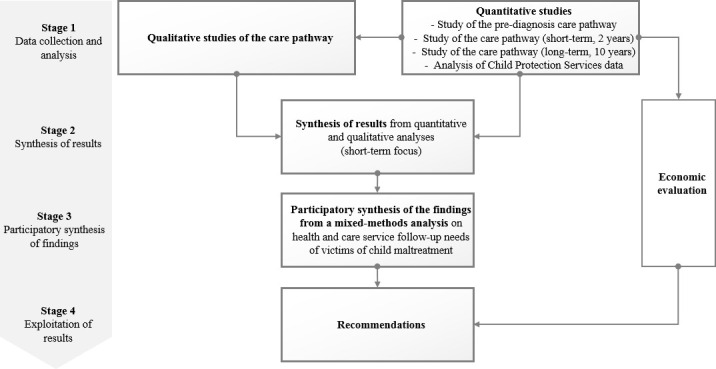
Interconnected Studies from the SERENA Collaborative Project

SERENA is a large-scale, Horizon Europe-funded research initiative involving 22 partners from multiple European countries (Figure 1), sectors and professions. The project period is from Jan 1, 2025 to Dec 31, 2029. It is coordinated under the standard Horizon Europe framework, with governance overseen in France. The project employs a cross-sectoral, mixed methods, multi-study design (Figure 2) comprising two scoping reviews, several nationwide quantitative studies from longitudinal HSC data, a multidisciplinary qualitative study, and a study of the societal costs of CM. These analyses will inform each other so that the findings from the studies can be combined and further analysed to yield an expanded understanding of service availability, provision, benefits and costs. The framework for analysis will involve consultations with HSC professionals and ASCM to ensure that interpretations, insights and recommendations are inclusive, evidence-based, and multifaceted.

Austria: Medical University of Vienna.

Denmark: Aarhus University, Nordsjaellands Hospital, ROCKWOOL Foundation.

France: CHU Dijon, France Europe Innovation, INSERM, INSERM Transfert, ORS BFC, Université de Bourgogne.

Germany: University Clinics Ulm.

Greece: Institouton Ygeias Tou Paidiou.

Ireland: Technological University Dublin, Tusla Child and Family Agency.

Netherlands: Erasmus School of Health Policy & Management.

Portugal: Lusófona University of Porto.

Romania: Babeş-Bolyai University.

Sweden: Stockholm University.

Switzerland: Zurich University of Applied Sciences, School of Social Work.

United Kingdom (England, Wales): Cardiff University, Swansea University Medical School, University College of London.

The project employs a cross-sectoral, mixed methods, multi-study design comprising several nationwide longitudinal quantitative studies, a qualitative study, and a study of the societal costs of CM. These findings and the participatory analysis and interpretation involving HSC professionals and ASCM will lead to evidence-based recommendations.

### Methodology: A Three-Step Process

**Figure 3:**
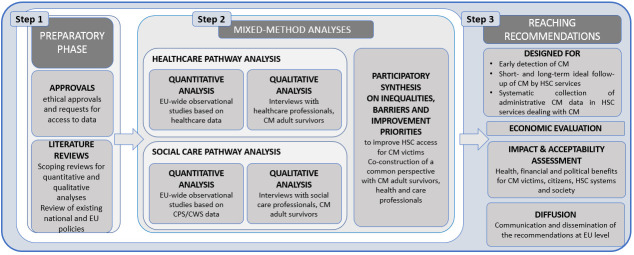
SERENA’s Three-Step Process

SERENA follows a three-step process, outlined in Figure 3:

The project follows a sequential three-step approach, which outlines the process from initial scoping reviews and data collection to mixed-method analyses and the development of evidence-based recommendations.

CM: child maltreatment; HSC: health and social care; CPS: child protective services; CWS: child welfare services.

#### Step 1

The preparatory phase: country selection and scoping reviews to refine criteria for the subsequent quantitative and qualitative studies.

The selection of countries for SERENA is informed by the methodologies and insights developed during the Euro-CAN project (www.euro-can.org). The quantitative longitudinal studies on healthcare pathways of maltreated children will include seven countries: Austria, Denmark, France, Germany, Sweden, Switzerland, and Wales. We will also conduct a longitudinal study examining the pathways through different administrative systems for children in care by linking health, education and social care datasets in Denmark and Wales. These countries were selected as they have already linked these datasets and made them accessible to researchers. This will allow us to examine trajectories over time, transitions between different systems and longer-term health, social care and educational outcomes from childhood into adolescence and early adulthood. In addition, we will supplement this analysis with the ROCKWOOL-Duke Global Child Welfare Database [19], which aggregates cross-national data on children in welfare systems across 26 countries in Europe. In the absence of a centralised national medico-administrative database on CM data in Austria, data will be sourced from the FOKUS database, a regional tertiary hospital-based child protection unit in Vienna, which represents an umbrella service for all hospital-based CP teams in the region [20]. The qualitative study will include data from Denmark, France, and Wales, the findings from which can be combined with findings from the quantitative studies to give a comprehensive life course approach. Interviews with ASCM and HSC professionals in these countries will explore access to HSC services for maltreated children.

For the quantitative studies, CM will be grouped in three categories: (1) physical abuse, identified via an existing algorithm based on predefined diagnosis codes, according to the International Classification of Diseases 10th Revision (ICD-10); (2) sexual abuse; and (3) psychological abuse, or neglect, for which we will develop new ICD-10-based algorithms through scoping reviews and existing algorithms from Euro-CAN. We have already demonstrated the feasibility of developing such algorithms and carrying out pooled analyses in our initial work on physical abuse [10].

We will conduct two scoping reviews. The first scoping review [21] will examine barriers and facilitators to accessing HSC therapeutic services for children who have experienced child maltreatment and will be conducted in two parts. A review of qualitative studies will include literature from France, Denmark, and the UK. The review of quantitative studies will incorporate 12 countries: Austria, Denmark, France, Germany, Greece, Ireland, the Netherlands, Portugal, Romania, Sweden, Switzerland, and the UK. The findings will inform the designs for the qualitative and quantitative studies. The second scoping review will examine the pathways that children with maltreatment take through services, including health, social care, education and other services. The review will help to complement our analysis conducted in Denmark and UK and on the ROCKWOOL dataset, by situating the findings in a broader international context.

#### Step 2

Mixed methods analyses involving quantitative and qualitative studies to assess how maltreated children navigate HSC services. A final synthesis of study results will be reviewed by an expert group of ASCM and HSC professionals.

The quantitative studies of healthcare pathways will utilise nationwide medico-administrative data from six countries (Denmark, France, Germany, Sweden, Switzerland and Wales) and the FOKUS regional tertiary hospital database from Austria to compare the healthcare pathways of maltreated children with those not identified as exposed to maltreatment, across three time points: before maltreatment diagnosis, short-term follow-up to two years, and long-term follow-up to ten years. We will use different algorithms to identify each type of abuse in medical-administrative databases. For physical abuse, we will use the algorithm already developed as part of the Euro-CAN project [10], based on ICD-10 codes used to report physical abuse and specific to intentional physical assaults. For other types of abuse, we will develop algorithms in collaboration with all partners. To this end, as for physical abuse, we will organise several meetings to take into account the specific coding practices in each country. To account for these differences, we will use a minimal set of ICD-10 codes available in all participating countries, which will allow us to apply a standardised approach to carry out the planned analyses. The age considered will vary based on the type of maltreatment. Specifically, physical abuse is more reliably identified before the age of walking, leading to the inclusion of children less than two years of age. Conversely, sexual or psychological abuse, and neglect will be retrieved for children up to 17 years old. For each maltreatment type, we will describe characteristics at the index hospitalisation by gender, age, diagnoses and procedures, length of stay, intensive care unit admission, and in-hospital death.

We will identify any prior hospitalisations before the diagnosis of CM identified between 2013 and 2022. This retrospective study will be carried out between 1 January 2025 and 31 December 2026. For physical abuse, this will include birth data such as gestational age, birth weight, small for gestational age, and foetal sex, as well as neonatal conditions such as respiratory disorders and congenital malformations following EUROCAT guidelines [22]. For sexual abuse, psychological abuse, and neglect, we will use data on hospitalisations within four years before the index hospitalisation, including the number of hospitalisations, length of stay, diagnoses, and procedures.

For these previously identified CM children, we will then study the number of hospitalisations, maltreatment readmissions, diagnosed physical and mental health conditions (e.g. asthma, neurological disorders, suicidal behaviours, anxiety), and in-hospital deaths, at short-term follow-up (2 years) and long-term follow-up (10 years). These retrospective studies will be carried out between 1 January 2026 and 31 December 2027 for the short-term and between 1 January 2027 and 31 December 2028 for the long-term. Denmark, France and Wales have access to outpatient data which will enable the assessment of consultations with general practitioners and specialists, redeemed prescriptions for drugs, deaths, and socio-economic status.

For all the quantitative studies mentioned above, we will consider a matching approach (1:3) to compare maltreated children and children not identified as maltreated, matched mainly on date of index hospitalisation, age at index hospitalisation, gender, and region. The analyses will use identical methods across all countries, and the results will be pooled and weighted by the number of maltreated children per country.

The quantitative studies of social care pathways will use linked individual level child protective services (CPS) registry data from Denmark and Wales to map children’s pathways through HSC systems between 2000 and 2025, comparing maltreated children known to social care services with children not identified as maltreated. This retrospective study will be carried out between 1 January 2026 and 31 December 2027 These registries include demographics, in- and outpatient hospital contacts and services, general practitioner visits, child protection, education, and justice data. We will conduct analyses by sector, and stratified by age, follow-up time, and country. For other countries, we will use aggregate statistics from the ROCKWOOL-Duke Global Child Welfare Database, which includes data on CM investigations, confirmed cases, and out-of-home care data.

The qualitative study of HSC pathways will be based on individual and group interviews with HSC professionals and interviews with ASCM (work to be carried out between 1 January 2025 and 31 December 2027). Participants will be recruited via existing networks and open recruitment, provided with study information, and asked to give written informed consent. Data collection will be conducted in 2025 - 2026 by trained interviewers in local languages. Data collection will be informed by findings from the scoping reviews and public consultations (e.g., with the Welsh advisory group of young people with lived experience of social care services (CASCADE Voices)). In line with the wider project, CM will be defined as acts of commission and omission (neglect) by permanent or temporary caregivers and exclude victimisation by non-caregivers such as strangers, acquaintances, and peers [17, 23]. We will analyse transcripts using thematic analysis, drawing on a mix of pre-specified themes (e.g. barriers to access for available services) and by identifying themes within the data.

In the final stage of Step 2, we will produce a cross-case synthesis using a convergent mixed methods design [24]. We will thematically analyse data from the findings of the qualitative and quantitative studies on short-term follow-up after CM identification guided by the frameworks of Boyatzis [25], and Braun and Clarke [26]. The discussion of the analysis will reference the findings of scoping reviews. We will then conduct an interdisciplinary, participatory synthesis, involving HSC professionals and ASCM, to interpret and assess the facilitators of, and barriers to, HSC services and to identify priority actions. Building on the Euro-CAN network, SERENA will establish an ASCM Board, composed of six diverse members selected via snowball sampling. The board will contribute to project design and interpretation of findings, with members receiving training, support, and compensation.

The initial synthesis of findings will be shared with the ASCB board through individual interviews, using an expert guide to minimise bias. Feedback will guide further analyses, overseen by a board member, with updates provided through online exchanges. Discrepancies between academic and survivor perspectives will be actively discussed and documented. The approach will inform the final analysis to identify patterns, confirmatory and discordant findings from the studies and identify priority actions across countries. A revised synthesis will be reviewed by the board, with two virtual meetings and a final in-person meeting. This participatory approach will ensure the recommendations are credible and informed by survivor expertise.

#### Step 3

Reaching Recommendations

We will use the findings of the studies to develop evidence-based recommendations for improving detection, intervention and follow-up, and administrative data collection on CM. These recommendations, aimed at national and European policymakers and HSC professionals, will be based on the results of the mixed methods analysis, societal cost estimates, and input from ASCM and HSC providers, to ensure impact and acceptability.

The estimate of the societal costs of CM will include short- and long-term HSC costs and productivity losses. This economic evaluation will allow us to compare implementation costs with expected health and social benefits. Estimates will be based on linked hospital, social care, and educational data, following standard guidelines for economic evaluations [27].

Although data availability varies by country, a comparative analysis approach will be used to estimate direct healthcare costs, other sectoral costs, and indirect costs from productivity losses associated with CM. Direct costs will be calculated by combining healthcare and social care utilisation data multiplied by relevant unit costs. In addition, education and productivity losses will be included to reflect the broader societal impact of CM. We will estimate productivity losses using both the Human Capital Method and the Friction Cost Method [28]. Cost estimates will use non-parametric bootstrapping due to skewed distributions. Multiple regression models will be conducted to address the evaluation of potential effect modifiers such as age, gender, education, and urbanisation. First, average costs for non-identified maltreated children will be estimated using a weighted intercept-only generalised linear model, providing baseline insights into overall cost patterns. Secondly, the impact of each maltreatment type will be assessed by regressing individual maltreatment variables against costs. Finally, population-level costs will be extrapolated by multiplying the adjusted annual per-person excess costs by the prevalence of maltreatment exposure in the population.

## Discussion

SERENA is a large-scale research initiative funded by Horizon Europe, designed to enrich the current body of knowledge regarding CM and the role of HSC services and improve evidence-informed practices. The project comprises 22 partners from multiple European countries and operates within the Horizon Europe framework, with governance based in France. Using multi-country, multi-sectoral, mixed methods, and a longitudinal design, SERENA aims to provide evidence-based recommendations for HSC professionals and policymakers to enhance CM detection and optimise the planning and delivery of HSC services. The project’s novelty is its comprehensive approach, as the recommendations will: (1) be based on a European-wide project on HSC access and barriers for maltreated children; (2) draw on both quantitative and qualitative data from multiple countries; (3) include an economic evaluation of the societal costs of CM; (4) be developed through a participatory process involving key stakeholders including ASCM; and (5) be tailored to national, sector-specific, and European level contexts, including guidance on capacity building and training to improve both short- and long-term outcomes for children affected by CM.

## Strengths and Limitations

Epidemiological studies indicate a lack of research on assessing and improving access to HSC services for maltreated children, which affects decision-making, policy, and practice in child welfare and child protection services [29]. There is a lack of longitudinal, national, register-based studies on CM and HSC utilisation in low and middle-income countries. Even in high-income settings, longitudinal evidence remains scarce [29]. Our novel approach comprises a multi-country, cross-sectoral, mixed methods design which combines longitudinal administrative HSC data, qualitative case studies, and cross-national aggregated child protection data. This facilitates the generation of comprehensive insights into HSC utilisation and barriers to accessing services and addresses several gaps in the evidence. An additional strength is the use of administrative HSC data, enabling analysis of pooled individual level and cross-sectoral data across countries. This real-world project will utilise nationwide data from several European countries, allowing large-scale examination of CM and HSC services with sufficient numbers of recorded children with CM for robust outcome analyses.

SERENA will conduct a multinational longitudinal retrospective cohort study using social- and health care linked registry data. The inclusion of longitudinal data, covering a period of up to 10 years or more, with a recorded timing of maltreatment event will provide valuable insights into early life trajectories of affected children, enhancing our understanding of how experiences during potentially sensitive developmental periods can influence pathways in health and wellbeing.

This design addresses a notable gap in the literature, as most studies on maltreated children’s health rely on retrospective surveys of adults with medical conditions, which are prone to recall bias and false negatives [7, 30]. Longitudinal studies using administrative data can overcome these limitations [7]. In our longitudinal study of pathways through services for maltreated children in care, we will link individual level social care data from Denmark and Wales with demographic, health, education and justice sectors data. This will allow us to map children’s trajectories through HSC systems, analyse transitions between systems, and identify patterns and barriers to access to HSC services. We will supplement this analysis with the ROCKWOOL-Duke Global Child Welfare Database, which aggregates harmonised data from child welfare and statistical agencies across 26 European countries. Before the submission of this European SERENA project, most longitudinal CM studies were US-based.

Compared to using siloed databases to explore HSC responses to CM, our use of cross-sectoral administrative data linkage enables us to account for the way multiple interacting factors influence outcomes across critical periods in people’s lives [27] and to systematically examine population-level responses to CM across multiple and overlapping domains [31, 32]. The participation of ASCM and HSC professionals further ensures that evidence and recommendations cut across systems and reflect people’s lived experiences [27]. In these ways, our approach advances methodological practices in cross-sectoral life course research in Europe and supports efforts to redesign systems that improve health and wellbeing trajectories across the lifespan [4].

SERENA has limitations. Cultural perceptions of CM may lead to different understandings of the qualitative interview questions. There may be challenges with reporting abuse-related service experiences due to stigma and recall issues. We will attempt to mitigate these issues through the use of trained and experienced interviewers and multiple participant recruitment strategies. In the quantitative data, national variations in coding practices for CM types will be addressed by applying algorithms for physical abuse from a previous multinational European study [10] and by developing new algorithms for other types of abuse that account for cultural differences in definitions and recording.

Exposure misclassification due to under-reporting and under-identification of all types of CM will be a feature of administrative data from all sectors, likely leading to an underestimation of the true prevalence. In addition, medico-administrative data captures data on the children most severely affected by CM, representing the “tip of the iceberg” of the phenomenon. While this may limit generalisability to the broader population, our intention in identifying early life course HSC pathways and outcomes for children who experience CM is to help understand the downstream and later life consequences of CM and discuss the effects of access or barriers to HSC services. We will combine our insights with the economic evaluation to recommend upstream policy and practice changes that support effective allocation of our collective resources to benefit children and young people who experience all forms of CM and who have contact with our HSC services [33].

Aligned to this objective and in recognition of the specific limitations of medico-administrative data, we will link social care data with health data. This will make it possible to identify children involved with social care services who have not been to hospital, and to study the care pathways of children identified by either sector. Given that a large proportion of CM-affected individuals are not reported to child protection services [34], some misclassification should still be expected. However, by pooling individual level data across several European countries, SERENA enables the study of CM in one of the largest populations to date. This increases statistical power and makes it possible to detect associations that may previously have gone undetected. To further minimise bias and improve representativeness and interpretive robustness, findings will be synthesised with qualitative results and reviewed by HSC professionals and ASCM stakeholders, who can also recommend additional analyses. Another limitation is that the algorithms used to identify CM types do not explicitly capture the timing or frequency of maltreatment. CM often occurs covertly and may go undetected for long periods, if identified at all. However, the fact that SERENA exploits the databases’ capacity to link healthcare episodes over time on an individual level will enable the identification of recurrent events and patterns among identified children, offering unique insights into recurrence and potential cumulative harm. Nevertheless, the data remains limited to the subset of children identified through HSC administrative data.

System-level challenges in the identification and response to CM may result from limited training among health professionals [32], uncertainty about legal procedures, and concerns about the consequences of misreporting [35]. Coordination and information-sharing across HSC services and judicial systems can be complex and fragmented [17, 36], potentially hindering timely and effective responses.

Moreover, the recognition of maltreatment does not necessarily ensure quick access to support [32], due to potential workforce shortages and long waiting times for mental health services. While these challenges are well recognised in the field, they have received limited attention in cross-sectoral and multinational research. SERENA may be in a unique position to address these gaps by examining barriers to identify maltreatment and access care from both quantitative and qualitative perspectives.

### Expected Results

SERENA expects to improve access to HSC services for maltreated children by generating robust, multidisciplinary evidence on HSC pathways across Europe and developing European level recommendations to enhance detection and intervention, ultimately improving short- and long-term outcomes for individuals and society.

We expect that the two scoping reviews will map existing HSC pathways for maltreated children and provide insights to refine the subsequent mixed-method studies. In addition, we will develop validated algorithms for identifying physical abuse, sexual abuse, psychological abuse, and neglect in administrative health records, facilitating cross-country comparability and strengthening future research on CM.

From the multi-study mixed-method analyses, we expect to provide a clearer picture of HSC pathways before and after CM identification, thus highlighting existing gaps in access and follow-up. We hypothesise that the quantitative studies will find increased morbidity and more frequent hospital visits among maltreated children compared to the general population, both for acute and chronic conditions. Furthermore, we expect to observe patterns of increased healthcare utilisation, help-seeking behaviour, and social care use. Additionally, we hypothesise that early predictors of CM can be identified in HSC data, along with the typical age of identification. The qualitative study is expected to generate in-depth insights into how HSC services for children who experience CM are currently implemented, and how access to these services can be facilitated. Finally, we expect the economic evaluation to reveal an increased financial burden associated with CM, compared to the general population, on a societal level. By incorporating both healthcare and educational costs, we expect to contribute to a more comprehensive understanding of the broader societal impact of CM. Thus, we expect our analysis to provide robust insights into the complex interplay between CM and its short- and long-term outcomes as well as the temporal benefits of HSC interventions in mitigating its negative effects [1, 4, 37].

Ultimately, we expect that the findings and participatory analysis and interpretation involving HSC professionals and ASCM will lead to evidence-based recommendations that can be implemented by decision-makers for public health policy. The key objectives of these recommendations will be to reinforce planning, delivery, and early detection of CM across HSC systems throughout Europe. As a result, evidence-based practices and research in this area will be enhanced, HSC services will be mapped out, and additional intervention services and management strategies will be offered to children and young adults who have been maltreated.

### Expected Implications

Based on findings from multiple sources within the SERENA studies, including the economic evaluation of societal costs, we will develop recommendations for policymakers at the European level. These recommendations will emphasise the avoidable costs of CM to society, and promote investment in evidence-based policies and practices for improved detection, intervention, follow-up, and systematic data collection on CM within HSC services. They will also address the introduction of necessary legal provisions, the promotion of multi-sectoral coordination, the creation of safe environments in HSC services, and the capacity building of frontline professionals, in alignment with the INSPIRE programme, a set of seven evidence-based strategies for countries and communities working to eliminate violence against children, developed by WHO and partners [38].

The results are expected to provide crucial baseline evidence on children who experience CM prior to key life transitions, during which critical and formative periods occur. For example, the transition into adulthood is a stage when prolonged absence from education, social engagement, or support systems can have lasting adverse effects on labour market participation as well as physical and mental health. Early life adversities, such as maltreatment, may disrupt brain maturation, physiological growth, and social adaptation, increasing the risk of negative health, social, and educational outcomes across the life course. Children and adolescents who have experienced maltreatment are a population at risk, and this early adversity can disturb developmental trajectories, increasing the risk of disengagement from education or employment and other long-term negative outcomes.

The findings from SERENA are expected to have relevance beyond Europe by providing transferable evidence on detection algorithms, critical developmental periods, service coordination, long-term health outcomes, and health behaviour patterns. These insights can guide policymakers and practitioners to adapt detection, intervention, and prevention strategies to local contexts, including high-, middle-, and low-income settings, even where resources or data infrastructure are limited. By highlighting children’s trajectories prior to key life transitions, SERENA will inform prioritisation of early interventions, multi-sectoral collaboration, and cost-effective approaches to mitigate the long-term consequences of CM.

At the European level, SERENA will guide sectoral, national, and European policies to reduce disparities and strengthen early CM detection and intervention within HSC services.

Evidence to support targeted early CM detection and intervention aligns with the World Health Assembly’s public health approach to delivering comprehensive care throughout the life course [39]. The results will guide the development of standardised approaches to data collection and service provision, potentially enabling more timely and equitable responses to CM in Europe.

Although the substantial burden of CM is widely recognised, its societal costs remain poorly quantified and current evidence is dated. According to the WHO [40], these costs are likely in the tens of billions of euros in Europe, with healthcare accounting for a significant portion [37]. For instance, Conti et al. [14] found that short-term somatic and mental health issues in the UK comprise over 40% of CM-related costs. Additionally, CM leads to increased risk of substance abuse, school and work absence, unemployment, and intergenerational violence, contributing to wider social and economic consequences [8, 41]. SERENA will address this critical knowledge gap by providing current, cross-country estimates of these costs. This economic analysis will support decision-makers in understanding the long-term societal impact of CM and strengthen the case for investing in effective, preventive, and coordinated care systems, with potential relevance for diverse income settings globally.

### Project Feasibility

SERENA is funded by Horizon Europe, Grant Agreement ID: 101151854, involving 22 partners from 12 countries and operating under the standard Grant Agreement protocol with structured work packages and strict reporting times.

The project spans 2025–2029 and follows a three-step process that encompasses scoping reviews, quantitative studies, qualitative research, and economic evaluation. While work package interdependence means delays in one component may affect subsequent studies, this risk will be mitigated through strategic planning, interim deadlines, and close collaboration to ensure progress and knowledge transfer. All participating countries are Euro-CAN partners with confirmed data access, strengthening the feasibility of the project and ensuring consistency across national datasets. SERENA builds on strong institutional collaboration and methodological expertise, ensuring scientific rigour and project deliverability.

## Conclusion

SERENA will advance the state-of-the-art in the field of CM by generating robust, multi-country evidence regarding CM and related use of HSC services across Europe. The project’s cross-sectoral and mixed methods approach should considerably improve our understanding of the barriers to service access, allow us to estimate the societal costs of CM and prioritise future actions informed by lived experience and professional expertise.

Our findings will support evidence-based recommendations for early detection, improved service pathways, and policy development on national and European levels. These recommendations will aim to strengthen HSC system responses, promote cross-sector collaboration, and ultimately to improve outcomes for maltreated children and society at large.

Importantly, the evidence generated by SERENA is designed to be transferable beyond Europe, providing insights and methodological frameworks that can inform policy and practice globally, across different economic settings.

## Data Availability

In accordance with the rules of the European Commission and the consortium agreement signed between the SERENA project partners, only published information may be shared.

## Abbreviations

CM:	Child Maltreatment
HSC:	Health and Social Care
ICD-10:	International Classification of Diseases 10th Revision
CPS:	Child Protective Services
WHO:	World Health Organization
